# Prenatal Exposure to Urban Air Nanoparticles in Mice Causes Altered Neuronal Differentiation and Depression-Like Responses

**DOI:** 10.1371/journal.pone.0064128

**Published:** 2013-05-29

**Authors:** David A. Davis, Marco Bortolato, Sean C. Godar, Thomas K. Sander, Nahoko Iwata, Payam Pakbin, Jean C. Shih, Kiros Berhane, Rob McConnell, Constantinos Sioutas, Caleb E. Finch, Todd E. Morgan

**Affiliations:** 1 Davis School of Gerontology, USC, Los Angeles, California, United States of America; 2 School of Pharmacy, USC, Los Angeles, California, United States of America; 3 Dornsife College of Letters, Arts and Sciences, USC, Los Angeles, California, United States of America; 4 Viterbi School of Engineering, USC, Los Angeles, California, United States of America; 5 Dept. of Neurobiology, Dornsife College, USC, Los Angeles, California, United States of America; 6 Keck School of Medicine, USC, Los Angeles, California, United States of America; 7 Dept. of Pharmacology and Toxicology, School of Pharmacy, University of Kansas, Lawrence, Kansas, United States of America; Virginia Commonwealth University, United States of America

## Abstract

Emerging evidence suggests that excessive exposure to traffic-derived air pollution during pregnancy may increase the vulnerability to neurodevelopmental alterations that underlie a broad array of neuropsychiatric disorders. We present a mouse model for prenatal exposure to urban freeway nanoparticulate matter (nPM). In prior studies, we developed a model for adult rodent exposure to re-aerosolized urban nPM which caused inflammatory brain responses with altered neuronal glutamatergic functions. nPMs are collected continuously for one month from a local freeway and stored as an aqueous suspension, prior to re-aerosolization for exposure of mice under controlled dose and duration. This paradigm was used for a pilot study of prenatal nPM impact on neonatal neurons and adult behaviors. Adult C57BL/6J female mice were exposed to re-aerosolized nPM (350 µg/m^3^) or control filtered ambient air for 10 weeks (3×5 hour exposures per week), encompassing gestation and oocyte maturation prior to mating. Prenatal nPM did not alter litter size, pup weight, or postnatal growth. Neonatal cerebral cortex neurons at 24 hours *in vitro* showed impaired differentiation, with 50% reduction of stage 3 neurons with long neurites and correspondingly more undifferentiated neurons at Stages 0 and 1. Neuron number after 24 hours of culture was not altered by prenatal nPM exposure. Addition of exogenous nPM (2 µg/ml) to the cultures impaired pyramidal neuron Stage 3 differentiation by 60%. Adult males showed increased depression-like responses in the tail-suspension test, but not anxiety-related behaviors. These pilot data suggest that prenatal exposure to nPM can alter neuronal differentiation with gender-specific behavioral sequelae that may be relevant to human prenatal exposure to urban vehicular aerosols.

## Introduction

Environmental influences on neurodevelopmental disorders are well recognized [1], [2]. Emerging evidence from epidemiologic studies implicates air pollution exposure as a risk factor for neurodevelopmental disorders including autism-spectrum disorders (ASD) [3], [4], anxiety-spectrum disorders, depression, and schizophrenia [5], [6], [7]. In particular, air pollutant exposure during pregnancy and early life was associated with higher risk of ASD [4], of attention-deficit hyperactivity disorder [8], of anxiety and depression [9], and of schizophrenia [10]. For example, the risk of ASD was about 2-fold higher from maternal residence within 309 meters of freeways in California [4] and was similarly elevated with exposure during the first postnatal year [3]. Moreover, impaired cognitive functions and white matter abnormalities in later childhood were associated with severe urban air pollution [11]. Although the developing brain has recognized sensitivity to prenatal exposure to diverse air pollutants [12], [13], [14], the neurobiology of responses to vehicular emissions is largely unknown.

Rodent models employing direct maternal inhalation to diesel exhaust showed various postnatal consequences, including modestly decreased spontaneous locomotor activity and increased anxiety [15], increased glutamatergic NMDA receptors [16], region-specific alterations in catecholamine metabolites [17], [18], microglial activation [15], and apoptosis in cerebellar Purkinje neurons [19], [20]. Prenatal exposure to diesel particulates by maternal oropharyngeal aspiration increased IL-6 in fetal brains [15] and in the placenta [21]. How these effects are transmitted from the maternal lung to the placenta and fetal brain is obscure. To achieve more controlled and generalizable exposure than direct exposure to freeway air or to exhaust from laboratory combustion engines, we utilized novel technology in which airborne particulate matter from urban traffic in the Los Angeles Basin is collected over extended times and stored in aqueous suspension for re-aerosolization [22]. We focus on nanoscale (<200 nm) particulate matter (nPM), which has shown the most toxicity in rodent brain and vascular tissues [23], [24]. Exposure of adult male mice for 150 hours distributed over 10 weeks induced brain inflammatory cytokines and selectively altered glutamate receptor subtypes [25], [26]. These glutamatergic changes have potential relevance to ASD and other neurodevelopmental disorders in which glutamatergic functions are implicated [27], [28].

The present pilot study evaluated the applicability of the re-aerosolized prenatal nPM exposure paradigm to evaluate prenatal effects on brain development and its adult neurobehavioral sequelae. Neurons were cultured from neonatal cerebral cortex and observed for effects of prenatal nPM exposure on neuronal differentiation during the first 24 hours *in vitro*. Adults were later examined for behaviors that are implicated in neurodevelopmental disorders.

## Materials and Methods

### nPM Collection

Vehicular-derived nanoscale particulate matter (nPM <200 nm) was collected continuously for a 30-day period in March 2010 from a site nearby the downtown Los Angeles, CA I-110 Freeway on pre-treated Teflon filters (20×25.4 cm, PTFE, 2 µm pore; Pall Life Sciences) using a High-Volume Ultrafine Particle (HVUP) Sampler at 400 L/min [22], [25], [29]. Nanoparticles were transferred from the filters into sterile aqueous suspension by sonication. Aqueous suspensions of nPM were stored frozen as a stock at −20°C, which retains chemical stability for >30 days [30], including long-lived free radicals [25].

### Ethics Statement

Animal experiments were approved by the Institutional Animal Care and Use Committee at USC (protocol #11677), and followed the recommendations in the Guide for the Care and Use of Laboratory Animals of the National Institutes of Health. All rodents were treated humanely and with regard for alleviation of suffering. We did not obtain specific permission to collect particles from the air. No endangered or protected species were involved.

### Prenatal nPM Exposure

Mice were exposed to re-aerosolized nPM [25]. Female C57BL/6J mice (3 mo. old) were housed in a specific-pathogen free environment with Purina Lab chow and sterile water *ad libitum*. On each day of exposure, mice were transferred from home cages to sealed exposure chambers with airflow that ensured adequate ventilation and minimized animal dander, CO_2,_ and NH_3._ Re-aerosolized nPM were dried and delivered by a VORTAN nebulizer with compressed particle-free air at mass concentration levels approximating 350 µg/m^3^ [25]. Particle size and concentration were monitored by a scanning Mobility Particle Sizer (SMPS Model 3080, TSI Inc). Control mice received ambient filtered room air in parallel chambers. A total of 150 hours of exposure during 10 weeks was given at intervals of 5 hr/day and 3 days/week. The re-aerosolized nPM was indistinguishable from direct inhalation in glial activation [25, in supplement data].

Females (4 per exposure group) were exposed for 7 weeks before conception and continued through gestation up to 2 days before birth. This exposure interval was based on the 3–6 week duration of follicular maturation in mice [31], [32] and the duration of cyclophosphamide toxicity on primordial oocytes, a toxin that increases birth defects [33]. Paternals were not exposed to nPM.

### Primary Neuronal Culture

Cerebral cortex neuronal cultures were prepared from postnatal day 1 (PD1) mice (5 pups each, two litters per condition, nPM exposed and control air). Briefly, whole brains were dissected in Hank’s Balanced salt solution supplemented with 15 mM HEPES. Following dissection, cerebral cortex was dissociated using 0.25% v/v trypsin and 0.1% v/v DNase, followed by passage through 40 µm sieve [34]. Dissociated cells were seeded separately from each pup on glass coverslips coated with poly-D-lysine & laminin, at 20,000 cells/cm^2^ in Dulbecco’s Modified Eagle’s Medium (DMEM) supplemented with B27 (Invitrogen), at 37°C in 5% CO_2_ [25], [34]. This protocol with serum-free media favors neuronal>glial survival: after 24 h, neurons were>98% of cells. Postnatal sensitivity to nPM of neurite outgrowth *in vitro* was evaluated with 2 µg/ml nPM from the same batch used for inhalation, a concentration that inhibits neurite outgrowth through glutamatergic mechanisms [25].

### Neurite Initiation and Elongation Assay

After 24 h incubation, cultured neurons were fixed in 4% paraformaldehyde (phosphate buffered saline, pH 7.4) and immunostained for neuron-specific βIII-tubulin (1∶500, rabbit; Sigma Chemical Co.) and F-actin in lamellipodia and growth cones (rhodamine phalloidin, 1∶40; Molecular Probes). Three stages of neuronal differentiation were identified by morphology: *Stage 0*, pre-lamellipodia; *Stage 1*, lamellipodia present, but no neurites, defined as processes extending ≥10 µm from the cell body with immunopositivity for βIII-tubulin (green) and F-actin (red); *Stages 2–3*, greater neurite extension [35], [36]. Stages of differentiation were analyzed from 350 neurons sampled from16 fluorescent images of 750 µm^2^ per coverslip per experimental condition.

### Western Blots

Hippocampal tissue from postnatal day 3 (PD3) brains were homogenized in RIPA buffer (50 mM Tris-HCl, pH 7.4, 150 mM NaCl, 0.25% deoxycholic acid, 1% NP-40, 1 mM EDTA) (Millipore) supplemented with 1 mM PMSF, 1 mM Na_3_V0_2_, 10 mM NaF, Phosphatases inhibitor cocktails (2 and 3; Sigma) and Roche Complete Mini EDTA-free; Protease Inhibitor Cocktail Tablet (Roche). Homogenates were centrifuged at 10,000 g/10 min at 4°C. Supernatant protein was assayed by BCA (BioRad) using bovine serum albumin (BSA) as a standard. For Western blots, 20 µg of total protein in SDS buffer was electrophoresed on 8 or 10% SDS- polyacrylamide gels, followed by transfer to polyvinylidene fluoride (PVDF) membranes; PVDF membranes were blocked with 5% BSA for 1 hr and probed with primary antibodies overnight at 4°C (Table S1), followed by secondary antibodies (1∶10,000) conjugated with IRDye 680 (rabbit, LI-COR Biosciences, Lincoln, NE) and IRDye 800 (mouse, LI-COR). Protein bands were quantified by Odyssey V3.0 software (LI-Cor Biosciences). Neuronal specific βIII-tubulin (1;5,000, Sigma) and β-actin (1;10,000, Sigma) were loading controls.

### Behavioral Testing

Behaviors were evaluated at 8 months of age in both sexes (n = 5–10/group). Litter effects were minimized by selecting mice from 3 litters within each experimental group.

#### Preliminary physical assessment

Posture, gait, heart rate, breathing frequency and neurological reflexes (righting reflex, postural reflex, eye-blink reflex and whisker-orienting reflex) were examined [37].

#### Tail suspension test

Mice were suspended by the tail using medical tape 30 cm above the base [38]. Environmental light was kept at 300 lux. Videorecording for 6 min assessed duration of immobility, latency to immobility, and number of fecal boli.

*Open field and Elevated plus-maze* are described in [39].

### Statistical analyses

#### Behavioral

Normality and homoscedasticity of data distribution were verified by the Kolmogorov-Smirnov and Bartlett’s test. Parametric analyses employed one-way ANOVAs (for repeated measures or independent factors, as appropriate), followed by Tukey’s test with Spjøtvoll-Stoline correction for *post-hoc* comparisons. Nonparametric comparisons employed the Kruskal-Wallis test, followed by Nemenyi’s test for *post-hoc* comparisons, with significance at *P≤*0.05. Data is presented as the median ± inter-quartile range (IQR).

#### Neurite differentiation

Because of the small effective sample size (N = 5 mice per condition, 1 mouse per culture), we used a rank transformed non-parametric analysis of variance to assess the effect of nPM on the proportion of neurons in different stages of differentiation. This outcome was dichotomized by cell stage, comparing the proportion of fully differentiated cells in Stage 3 with the proportion in Stage 0, in Stage 0 and 1 combined (excluding Stage 2), and in Stages 0–2 combined. The proportion of pyramidal and non-pyramidal cells was also assessed for each exposure condition. Statistical tests were based on two sided hypotheses at 0.05 level of significance. These analyses used the Statistical Analysis System Version 9.2 (SAS Institute). Data are presented as median ± IQR.

#### Westerns

Single comparison nonparametric analyses used Mann-Whitney test (two tailed, Prism Ver 5.0, Graph Pad), with significance at *P*≤0.05.These data are presented as % change from the median ± IQR of filtered air controls.

## Results

### Prenatal nPM Exposure

Following 10-week exposure to re-aerosolized nPM, pregnant mice appeared healthy and well groomed, without respiratory distress. Maternal body weight, litter size, and birth weight were normal. As 8 mo. old adults, the prenatal nPM had normal body and brain weights and no gross organ abnormalities at necropsy.

### Neurite Development *in vitro*

**Figure 1:**
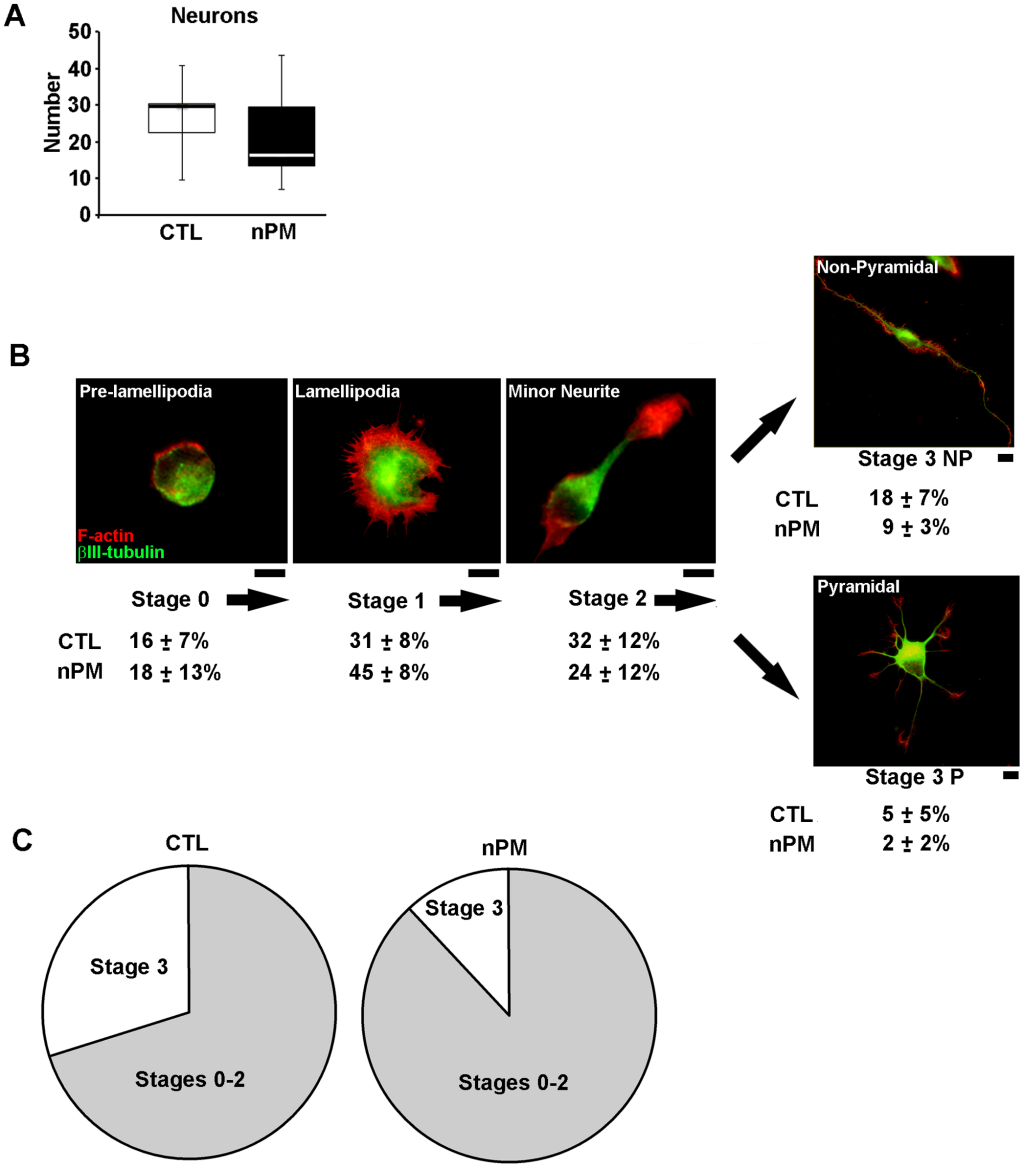
Prenatal exposure to maternally inhaled nPM impairs postnatal differentiation and neurite initiation in cultured neurons. Cerebral cortex neurons from day 1 pups were grown 24 hours in primary culture. a) Neuronal numbers (median±IQR) did not differ by prenatal nPM inhalation exposure (nPM) vs. filtered room air (CTL). b) Stages of neuron differentiation, green: βIII-tubulin and red: F-actin; scale bar, 10 µm. Prenatal exposure to nPM reduced the proportion of Stage 3 neurons with definitive neurite extension (Stage 3 non-pyramidal & pyramidal, with elongated neurites ≥15 µm)(median±IQR). c) Summary of prenatal nPM effects on neurite elongation: CTL Air, 30% of neurons had neurites ≥15 µm; nPM, 12% of neurons in nPM Air cultures (*P*<0.0001). N = 5 neuronal cultures/group.

Prenatal nPM exposure did not alter the numbers of neurons from cerebral cortex of neonatal mice after 1 day of culture (**Fig. 1A**).

The distribution of cells by stage of differentiation and, among elongated Stage 3 neurons with neurites ≥15 µm, the distribution into pyramidal and nonpyramidal neurons is illustrated in **Fig. 1B** with data for each exposure condition (prenatal nPM and control with and without exogenous postnatal nPM (2 µg/ml) exposure) in **Table 1**. There was no interaction between the prenatal nPM and exogenous nPM exposures on the proportion of Stage 3 neurons vs other stages, nor was there an effect of exogenous postnatal nPM on the proportion of Stage 3 neurons (nonparametric ANOVA). However, exposure to prenatal nPM altered the distribution of Stage 3 neurons (**Fig. 1C**) (*P*<0.0001). Corresponding to the decrease of Stage 3 neurons, there were 30% more Stage 0 and Stage 1 neurons, defined by the absence of definitive neurites, in the prenatal nPM exposed mice compared with controls (**Fig. 1B** **,** **Table 1**). Growth cones in Stage 3 neurons were 45% larger from prenatal nPM exposure (*P≤*0.05, not shown).

**Table 1:** Prenatal *in vivo* and postnatal *in vitro* nPM exposure by neuronal stage.

	CTL Litters		nPM Litters	
Stages of Neuritogenesis	*In vivo*(%)	*In vitro* nPM (%)	*In vivo* nPM (%)	*In vivo+In vitro* nPM (%)
**Stage 0: prelamellipodium**	17±1	15±6	24±13	17±5
**Stage 1: lamellipodium**	30±10	31±7	46±8	44±7
**Stage 2: minor neurite**	24±3	36±2	21±4	31±9
**Stage 3: Neurites (≥15 µm)**	24±11	20±7	13±6	9±3
**Stage 3: Pyramidal**	8±5	2±2	3±2	1±1
**Stage 3: non-pyramidal**	18±6	18±4	10±4	8±2

N = 5 cortical cultures/treatment; Median ± IQR.

**Figure 2:**
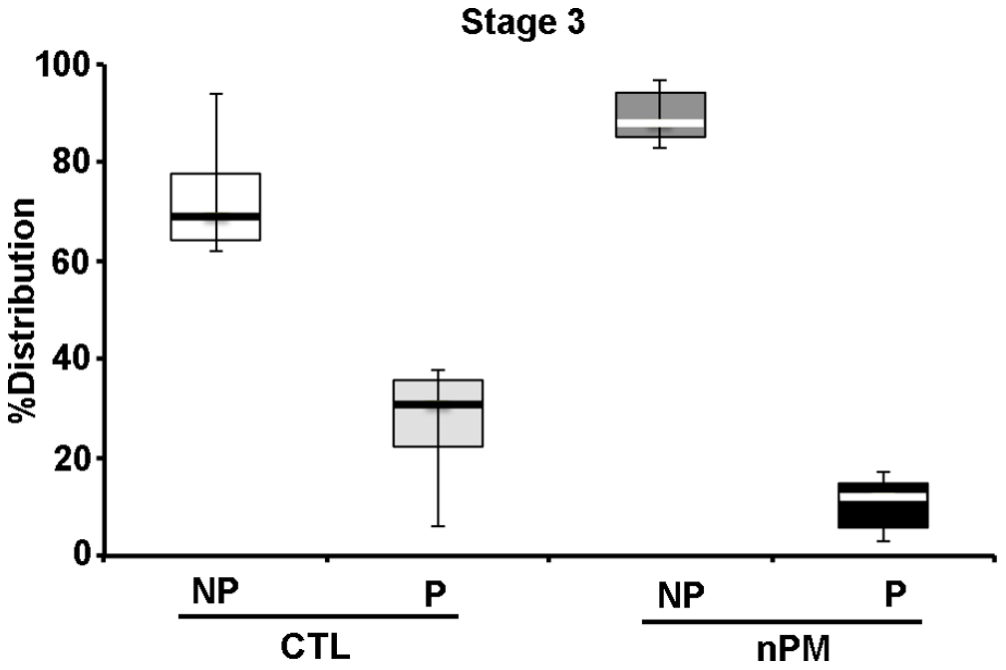
Postnatal effects of exogenous nPM on Stage 3 neurons *in vitro*. Data show the median proportions (± IQR) of pyramidal neurons (P) and non-pyramidal neurons (NP) from each postnatal cultures of nPM challenged cells (combining prenatal nPM and control air combined; N = 10 cultures/group). The proportion of Stage 3 pyramidal neurons to all Stage 3 types was reduced from 30% in replicate postnatal control cultures to 12% in cultures exposed to exogenous nPM (*P*<0.001).

Prenatal nPM did not alter the proportion of pyramidal vs. non-pyramidal neurons among Stage 3 cells. However, the proportion of Stage 3 pyramidal neurons to all Stage 3 types was reduced from 30% in replicate postnatal control cultures to 12% in cultures exposed to exogenous nPM (60% reduction, *P*<0.001) (**Fig. 2**).

### Proteins

Hippocampal lysates from neonatal mice were examined for protein markers representing glutamate receptors, neuronal growth cones, synaptic proteins, kinases and glial proteins (**Table S1**). The only significant change was a 30% decrease in JNK1 (c-Jun N-terminal kinase 1), but without change in its phosphorylation (**Fig. S1**). Because Met gene expression is impaired in some familial autism cases [40], we hypothesized that prenatal nPM exposure would also lower Met protein, but no change was found. Nor were changes found in GluA1, an AMPA glutamate receptor subunit that decreased during adult rat exposure to nPM [25] or in mGluR5, which is upregulated in autism [41], [42]. Ten other markers were unresponsive to prenatal nPM exposure, including IL-6 and other glial and inflammatory genes induced in adult mice by this exposure paradigm [15], [25].

### Behaviors

**Figure 3:**
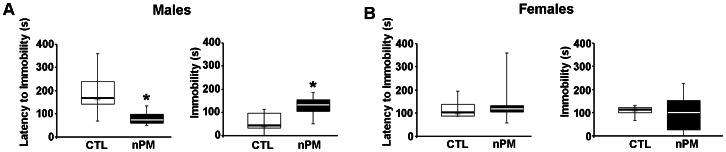
Prenatal nPM exposure and gender-specific adult behavior. Tail-suspension test. Males, but not females after prenatal nPM exposure exhibited depression-like responses in the lower latency prior to immobility and a longer immobility during suspension. However, nPM females were not affected. Data is expressed as median+IQR, *P*<0.05, N = 5–10 mice/group.

As adults of 8–10 months, the prenatal nPM exposed mice had normal posture, gait, heart rate, breathing frequency and neurological reflexes. The only behavioral alteration was in the tail suspension paradigm, wherein prenatally exposed males, but not females, had decreased latency, frequency, and duration of immobility (**Fig. 3**). There were no overt alterations in locomotor activity and anxiety-related parameters, as assessed by the open field and the elevated-plus maze paradigms, respectively (**Table S2**).

## Discussion

This pilot study indicates that prenatal exposure of mice to nPM from urban traffic can impair differentiation of neurons cultured from neonates and can alter gender-specific alterations of adult behaviors. This model is novel in two regards from prior studies: First, females were exposed for 7 weeks before mating to encompass the duration of oocyte maturation from primordial follicles. Second, we exposed mice to a specific size class of urban nPM (‘ultrafine PM’) collected continuously on filters from an urban freeway corridor (I-110) over 30 days, whereas other exposures were based on direct inhalation of ambient vehicular pollution or oro-pharyngeal infusion of suspended particles from a single diesel source (see Introduction). This roadway’s mixed vehicular traffic of gasoline and diesel engines is more representative of urban exposure than single source engines that are often used for rodent exposure studies. The nPM exposure of 350 µg/m^3^ approximated the high traffic levels in freeways impacted by heavy duty diesel truck traffic, 150–300 µg/m^3^ [43], [44]. The Los Angeles basin adult population is under intensive study and incurs high rates of vascular and pulmonary pathology [45]. Furthermore, two recent reports show increased prevalence of ASD in children living in close proximity to freeways [3], [4]. These preliminary findings corroborate and extend clinical and experimental evidence for the neurotoxic impact of prenatal exposure to air pollution and other environmental insults, which enhance vulnerability to ASD and other neurodevelopmental problems [13], [17], [46], [47]. These pilot findings, while of high translational potential, are presented cautiously because of the small sample size.

In this study, all mice appeared healthy and with no apparent alterations of neurological reflexes, posture and gait. Parturition and pup growth were normal. The litters were normal by pup number, neonatal brain and body weight, and postnatal growth. In contrast, others have reported impaired fetal somatic growth in association with behavioral deficits from prenatal exposure to diesel exhaust and other airborne PM [47].

Neuronal differentiation was impaired in cultures from neonatal cerebral cortex of prenatally exposed mice. After 24 hours in culture, we observed 50% fewer Stage 3 neurons, defined by neurites ≥15 µm. Neurons in Stage 3 also had larger growth cones. The addition of nPM to parallel cultures further impaired the differentiation of Stage 3 pyramidal neurons. Similarly, embryonic day 18 rat cerebral cortex neurons also responded to exogenous nPM with impaired differentiation of Stage 3 pyramidal neurons (unpublished observation). Analysis of these brains for neuronal populations is ongoing.

We hypothesize that the effects of nPM on pyramidal neuron differentiation *in vitro* will be rescued by glutamatergic agonists, because neurite outgrowth in pyramidal neurons is blocked by nPM and rescued by glutamatergic mechanisms (NMDA antagonist) [25] and because in spinal neurons, neurite initiation is also mediated by NMDA mechanisms [48]. Proximal mechanisms may include JNK- kinases (c-Jun-N-terminal kinases1–3), which mediate neurite extension in cortical neurons through microtubule stabilization [49], [50]. Neonatal hippocampal extracts of nPM exposed mice had lower JNK1, without change in phosphorylation of JNK1 (technical limitations prevented study of cerebral cortex). A JNK1 deficit would be consistent with altered neurite elongation, because phospho-JNK1 is concentrated in neurite tips [50]. Because JNK is ubiquitously expressed in glia as well as neurons, studies of homogenates cannot identify neuronal JNK. Inflammatory processes may also be involved *in vivo* from prenatal exposure, as observed in the IL-6 elevations of fetal brains at E17 after maternal exposure to diesel particulates [15]. The absence of IL-6 increases in the present neonatal hippocampus implies that prenatal inflammatory responses to vehicular particulates can be transient.

In the tail-suspension test, male adults from gestational nPM-exposure exhibited a longer duration and shorter latency of immobility. In mice, this index is posited to have high predictive validity for mental depression and low resilience to stress, because it is reduced by most antidepressant drugs [51], [52], [53]. Consistent with prior reports, we observed that females had higher baseline immobility than males [54]. This gender-based difference, which is postulated to reflect the higher prevalence of mental depression in women [55], may partially account for the lack of observed depression-like effects in nPM-exposed females, due to ceiling effects. In contrast, prenatal nPM did not affect anxiety-like responses in the novel open field or the elevated plus-maze. These data suggest that the long-term effects of prenatal nPM are limited to select domains of behavioral reactivity. Future testing will include assessment of social interactions, perseverative responses and cognitive integrity, more directly relevant to ASD-related manifestations.

Collectively, these findings suggest that transplacental influences from maternal nPM raise a threshold in the fetal brain for neuronal differentiation, which delays neurite initiation, but which does not impair neurite outgrowth once neurites are formed. The inhaled nPM may alter fetal development through placental inflammation, as observed in diesel particle exposure [15], [21]. Future analyses of neonatal and adult brain neuronal distribution and behaviors could reveal developmental retardation, which we anticipate will be subtle because of the overall normality of adult brain and behavior. The selective gender-specific behavioral changes are nonetheless relevant to ASD, which has been associated with pre-and postnatal exposure to vehicular derived air pollutants (Introduction). Although autistic human brains are characterized by excess (overgrowth) of cortical neurons in early childhood [56], later ages incur neurodegenerative changes in a third developmental phase of autism [57]. The present evidence for impaired or delayed cortical neuron differentiation might define an early stage of these impairments.

### Conclusions

These pilot findings of prenatal exposure to nPM are consistent with neurodevelopmental abnormalities in humans exposed to airborne pollution from vehicular traffic. These are also the first indications that nano-sized PM in air pollution has prenatal influences. We note that most of our analyses were performed using non-parametric analysis in recognition of the small sample size. While this might lead to missing detection of findings, it also gives credibility to the many significant effects that we have found. A caveat is the possibility of altered maternal behaviors from nPM exposure or potential changes in stress mediators during gestation. A subsequent study will address these concerns. Because of the evidence that closeness to freeway during gestation increases the risk of depression and other neurodevelopmental disorders, such as ASD, we present these preliminary findings with explicit caveats.

## Supporting Information

Figure S1 **c-Jun N-terminal Kinase (JNK) in neonatal hippocampal homogenates of prenatal nPM exposure.** Data from Western blots; nPM exposed and CTL air (N = 7 per group). a) Total JNK1 (c-Jun N-terminal Kinase 1) was reduced by −30% by prenatal nPM exposure (*P<*0.05). b) Total JNK2/3 c-d) Phosphorylated JNK1, 2 & 3.
(DOC)

Table S1 **Western blot data of neonatal hippocampal lysates from prenatal nPM exposure vs. filtered air controls.** Data presented is the % change from the median of filtered air controls. Significance is shown by bolding.
(DOC)

Table S2 **Behavioral outcomes of prenatal nPM exposure on open-field and elevated plus-maze behaviors in male and female adult mice (N = 5–10 mice/group).** *P≤0.05 CTL air vs. nPM; ANOVA; median ± I.Q.R.
(DOC)
