# Supplementary material for: Prenatal Exposure to Urban Air Nanoparticles in Mice Causes Altered Neuronal Differentiation and Depression-Like Responses
Source: PLoS One. 2013 May 29;8(5):e64128. doi: 10.1371/journal.pone.0064128 (PMC3667185; doi:10.1371/journal.pone.0064128)
Supplement: Figure S1 — c-Jun N-terminal Kinase (JNK) in neonatal hippocampal homogenates of prenatal nPM exposure. Data from Western blots; nPM exposed and CTL air (N = 7 per group). a) Total JNK1 (c-Jun N-terminal Kinase 1) was reduced by −30% by prenatal nPM exposure (P<0.05). b) Total JNK2/3 c-d) Phosphorylated JNK1, 2 & 3. (DOC) [file pone.0064128.s001.doc]

**
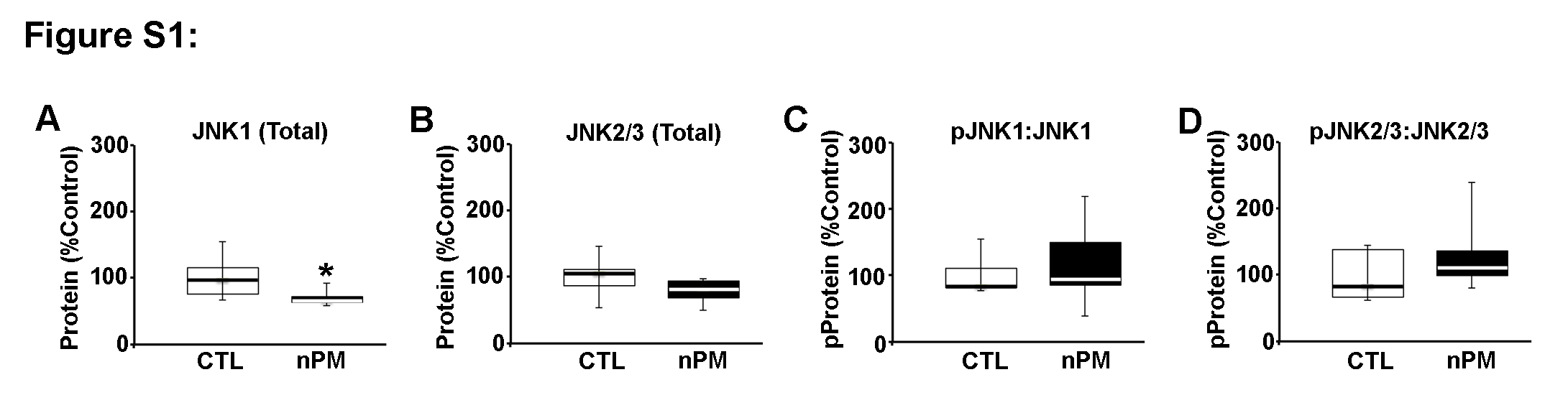
**

**Fig. S1: c-Jun N-terminal Kinase (JNK) in neonatal hippocampal homogenates of prenatal nPM exposure.** Data from Western blots; nPM exposed and CTL air (N=7 per group).  **a)** Total JNK1 (c-Jun N-terminal Kinase 1) was reduced by -30% by prenatal nPM exposure (*P<* 0.05). **b)** Total JNK2/3  **c-d)** Phosphorylated JNK1, 2 & 3.
