# Supplementary material for: Prenatal Exposure to Urban Air Nanoparticles in Mice Causes Altered Neuronal Differentiation and Depression-Like Responses
Source: PLoS One. 2013 May 29;8(5):e64128. doi: 10.1371/journal.pone.0064128 (PMC3667185; doi:10.1371/journal.pone.0064128)
Supplement: Table S1 — Western blot data of neonatal hippocampal lysates from prenatal nPM exposure vs. filtered air controls. Data presented is the % change from the median of filtered air controls. Significance is shown by bolding. (DOC) [file pone.0064128.s002.doc]

**Table S1: Western blot data of neonatal hippocampal lysates from prenatal nPM exposure vs. filtered air controls. Data presented is the % change from the median of filtered air controls. Significance is shown by bolding.**

|  |  |  |  |  |  |  |
| --- | --- | --- | --- | --- | --- | --- |
| **Antigens** | **% Change ± I.Q.R** | | ***P* value** | Species | **Dilution** | **Vendor** |
| ***General Synaptic Proteins*** | |  |  |  |  |  |
| PSD-95 | -23 | ± 14 |  | Mouse | 1; 1,000 | Abcam |
| Spinophillin | 18 | ± 29 |  | Rabbit | 1; 1,000 | Millipore |
| Synaptophysin | -10 | ± 15 |  | Mouse | 1; 5,000 | Millipore |
|  |  |  |  |  |  |  |
| ***Cytoskeleton & Growth Cone*** | |  |  |  |  |  |
| β-Actin | -13 | ± 30 |  | Mouse | 1; 10,000 | Sigma |
| βIII-tubulin | 0 | ± 4 |  | Rabbit | 1; 5,000 | Sigma |
| Capzβ2 | 4 | ± 11 |  | Mouse | 1; 1,000 | DHSB |
| Gap43 | -7 | ± 24 |  | Mouse | 1; 1,000 | Sigma |
| GFAP | 8 | ± 15 |  | Mouse | 1; 4,000 | Sigma |
| Tau46 | 10 | ± 20 |  | Mouse | 1; 1,000 | Sigma |
|  |  |  |  |  |  |  |
| ***Glutamate Receptors*** |  |  |  |  |  |  |
| **Ionotrophic** |  |  |  |  |  |  |
| GluA1 | -4 | ± 43 |  | Rabbit | 1; 1,000 | Abcam |
| pGluA1 Ser 831 | 16 | ± 8 |  | Rabbit | 1; 1,000 | Millipore |
| pGluA1 Ser 845 | -7 | ± 30 |  | Rabbit | 1; 1,000 | Millipore |
| GluA2 | -13 | ± 14 |  | Rabbit | 1; 1,000 | Millipore |
| GluN2A | -9 | ± 53 |  | Rabbit | 1; 1,000 | Millipore |
| GluN2B | 15 | ± 50 |  | Rabbit | 1; 1,000 | Millipore |
|  |  |  |  |  |  |  |
| **Metabotrophic** |  |  |  |  |  |  |
| mGluR5 100 kDA | -5 | ± 17 |  | Rabbit | 1; 1,000 | Abcam |
| mGluR5 250 kDA dimer | 3 | ± 15 |  | Rabbit | 1; 1,000 | Abcam |
| pmGluR5 Ser 996 | 7 | ± 10 |  | Rabbit | 1; 1,000 | Abcam |
|  |  |  |  |  |  |  |
| ***Kinases*** |  |  |  |  |  |  |
| **MAPK** |  |  |  |  |  |  |
| ERK1 (p44) | -2 | ± 19 |  | Rabbit | 1; 1,000 | Cell Signaling |
| pERK1 Thr 202 | 2 | ± 5 |  | Rabbit | 1; 1,000 | Cell Signaling |
| ERK2 (p42) | 9 | ± 10 |  | Rabbit | 1; 1,000 | Cell Signaling |
| pERK2 Tyr 204 | 2 | ± 5 |  | Rabbit | 1; 1,000 | Cell Signaling |
| P38 | 3 | ± 19 |  | Rabbit | 1; 1,000 | Cell Signaling |
| ***JNK1 (p46)*** | ***-33*** | ***± 7*** | ***P< 0.05*** | Rabbit | 1; 1,000 | Cell Signaling |
| pJNK1 Tyr 185 | 12 | ± 67 |  | Rabbit | 1; 1,000 | Cell Signaling |
| JNK2/3 (p54) | -23 | ± 25 |  | Rabbit | 1; 1,000 | Cell Signaling |
| pJNK2/3 Thr 183 | 32 | ± 39 |  | Rabbit | 1; 1,000 | Cell Signaling |
| ***JNK1:JNK2/3*** | ***-17*** | ***± 10*** | ***P< 0.05*** | Rabbit | 1; 1,000 | Cell Signaling |
| ***pJNK1:pJNK 2/3*** | ***-13*** | ***± 18*** | ***P< 0.05*** | Rabbit | 1; 1,000 | Cell Signaling |
| **Met Kinase** |  |  |  |  |  |  |
| Met (Total) | -1 | ± 36 |  | Mouse | 1; 1,000 | Cell Signaling |
|  |  |  |  |  |  |  |
|  |  |  |  |  |  |  |
|  |  |  |  |  |  |  |
