# Supplementary material for: Prenatal Exposure to Urban Air Nanoparticles in Mice Causes Altered Neuronal Differentiation and Depression-Like Responses
Source: PLoS One. 2013 May 29;8(5):e64128. doi: 10.1371/journal.pone.0064128 (PMC3667185; doi:10.1371/journal.pone.0064128)
Supplement: Table S2 — Behavioral outcomes of prenatal nPM exposure on open-field and elevated plus-maze behaviors in male and female adult mice (N = 5–10 mice/group). *P≤0.05 CTL air vs. nPM; ANOVA; median ± I.Q.R. (DOC) [file pone.0064128.s003.doc]

**Table S2. Behavioral outcomes of prenatal nPM exposure on open-field and elevated plus-maze behaviors in male and female adult mice (N=5-10 mice/group). **P* ≤ 0.05 CTL air vs. nPM; ANOVA; median ± I.Q.R.**

|  |  | **Males** | | **Females** | |
| --- | --- | --- | --- | --- | --- |
|  |  | **CTL** | **nPM** | **CTL** | **nPM** |
| **Open field** | |  |  |  |  |
|  | Total distance (cm) | 2711 ± 888 | 3436.76 ± 774 | 3535 ± 444 | 3493 ± 1083 |
|  | Time in center (s) | 247 ± 81 | 188 ± 109 | 221 ± 99 | 172 ± 90 |
|  | Percent activity in center (%) | 42 ± 15 | 41 ± 15 | 43 ± 9 | 43 ± 13 |
|  | Velocity (cm/s) | 5 ± 2 | 6 ± 1 | 6 ± 1 | 6 ± 2 |
|  | Fecal boli | 2 ± 4 | 2. ± 2 | 2 ± 4 | 3± 4 |
|  |  |  |  |  |  |
| **Elevated plus-maze** | |  |  |  |  |
|  | Open arm entries | 6 ± 3 | 8± 3 | 8 ± 2 | 9.± 6 |
|  | Closed arm entries | 7± 0 | 11 ± 3 | 11 ± 6 | 10 ± 4 |
|  | Total entries | 29 ± 6 | 36 ± 9 | 37 ± 18 | 40 ± 4* |
|  | Open arm duration (s) | 112 ± 94 | 132 ± 65 | 90 ± 17 | 120 ± 89 |
|  | Closed arm duration (s) | 140 ± 100 | 126. ± 72 | 179 ± 25 | 133 ± 77 |
|  | Fecal boli | 0 ± 0 | 0.± 0 | 0 ± 1 | 0.0 ± 1 |
